# Super-enhancement of 1.54 μm emission from erbium codoped with oxygen in silicon-on-insulator

**DOI:** 10.1038/srep37501

**Published:** 2016-11-22

**Authors:** M. A. Lourenço, M. M. Milošević, A. Gorin, R. M. Gwilliam, K. P. Homewood

**Affiliations:** 1 Materials Research Institute and School of Physics and Astronomy, Queen Mary University of London, Mile End Road, E1 4NS London, UK; 2 Advanced Technology Institute, Faculty of Engineering and Physical Sciences, University of Surrey, Guildford, Surrey, GU2 7XH, UK

## Abstract

We report on the super enhancement of the 1.54 μm Er emission in erbium doped silicon-on-insulator when codoped with oxygen at a ratio of 1:1. This is attributed to a more favourable crystal field splitting in the substitutional tetrahedral site favoured for the singly coordinated case. The results on these carefully matched implant profiles show that optical response is highly determined by the amount and ratio of erbium and oxygen present in the sample and ratios of O:Er greater than unity are severely detrimental to the Er emission. The most efficient luminescence is forty times higher than in silicon-on-insulator implanted with Er only. This super enhancement now offers a realistic route not only for optical communication applications but also for the implementation of silicon photonic integrated circuits for sensing, biomedical instrumentation and quantum communication.

The generation of efficient light emission in the 1.5 μm region is a subject of great interest in photonics research due to its importance for telecommunication applications. Silicon photonics is seen as a key enabling technology for the realization of highly integrated photonic circuits but the development of efficient light sources in silicon represents a serious challenge due to its indirect band gap. Several approaches have been attempted to achieve light emission in silicon^1^,^2^,^3^,^4^,^5^,^6^,^7^,^8^,^9^,^10^,^11^,^12^,^13^,^14^,^15^,^16^,^17^,^18^,^19^,^20^,^21^, including the incorporation of rare earth elements that led to luminescence at wavelengths characteristic of the rare earth internal transitions^22^,^23^,^24^,^25^,^26^,^27^,^28^,^29^. The rare earth Er, in particular, has been extensively studied due to its sharp luminescence at 1.54 μm, a wavelength corresponding to the absorption minimum in silica-based optical fibers. Optical communication applications were the main drivers for the early studies^30^,^31^,^32^,^33^,^34^,^35^,^36^,^37^,^38^,^39^,^40^,^41^,^42^,^43^,^44^,^45^,^46^,^47^,^48^ and luminescence at room temperature was demonstrated^37^,^38^,^39^,^40^, though quenching with temperature was observed.

The solid solubility of Er in Si is low (~10^18^ cm^−3^)^31^ and the use of non-equilibrium doping such as ion implantation and of codopants such as oxygen, fluorine or carbon at concentrations higher than that of Er have been shown to enhance the electrical and optical activation of Er ions^30^,^32^,^35^,^45^,^47^. In particular, for silicon codoped with oxygen and erbium, while there is a consensus that implantation of silicon with oxygen at concentrations greater than (around 10 times) that of the erbium^36^,^47^ is required for efficient electrical activation, the optimum O:Er ratio required to generate the maximum percentage of optically active Er ions is still not well established. Previous work reported on luminescence enhancement for O:Er carrier concentration ratios in the 1–10 range^30^,^35^,^36^,^46^,^48^. Hampering of the pumping of the Er optically active atoms has been attributed to the high concentration of precipitates and defects formed due to high implantation doses and also possible due to the high concentration of unpaired oxygen atoms that provide efficient non-radiative recombination routes^35^. In addition, in silicon layers codoped with Er and O grown by MBE, Er segregation at the surface was observed for O:Er ratio ≤1 while degradation of the quality of the epitaxial layer was observed for higher O concentrations (O:Er ratio ~ 16)^46^.

The rapid development of silicon nanophotonic integrated circuits^49^,^50^ has opened up many other applications for an erbium-silicon based light source, such as in sensing^51^ and biomedical diagnostics^52^. More recently, erbium doped materials have aroused renewed interest as a route to quantum communication technology owing to the atomic scale barrier to decoherence inherent in the rare earths due to the shielding of their *f*-shell levels. Multimode storage and retrieval of 1.54 μm photon has been demonstrated in Er^3+^:Y_2_SiO_5_^53^ and erbium-doped optical fibre^54^. Room temperature quantum bit storage exceeding 39 minutes using ionized donors in silicon has also been demonstrated^55^. Hence, the development of a 1.5 μm erbium doped silicon light source would interface with the emerging new technologies and current state of the art CMOS (Complementary Metal–Oxide–Semiconductor) which is based on an SOI platform. The rather narrow transmission window in SOI platforms, resulting from the short wavelength cut off (1.2 μm) due to the silicon band edge and the long wavelength limit (~2.4 μm) due to the onset of absorption in the buried oxide layer, makes the wavelength ranges typically used in optical communications still relevant.

Photoluminescence (PL) and gain measurements at 1.54 μm from silicon waveguides fabricated in SOI and implanted with erbium have been previously reported^56^. However, the gain values seen are close to the limit for the achievement of electrically pumped lasing, where the doping in the pn-junction leads to increased cavity losses due to free carrier absorption. Although the free carriers can be displaced from the main optical mode to reduce overlap to minimize these losses, increasing the fraction of optically active erbium would be highly, perhaps critically, beneficial. We report here on the 1.54 μm Er emission in erbium doped silicon-on-insulator when codoped with oxygen. Using carefully matched Er and O profiles, we show that the Er emission at 1.54 μm in SOI is maximum for equal oxygen and erbium volume concentrations, and is up to 40 times greater than in SOI samples without oxygen. The super enhancement on SOI structures codoped with Er and O is highly important as for the key applications such as lasers and optical amplifiers, Er needs to be incorporated into an SOI platform.

## Results and Discussion

**Figure 1:**
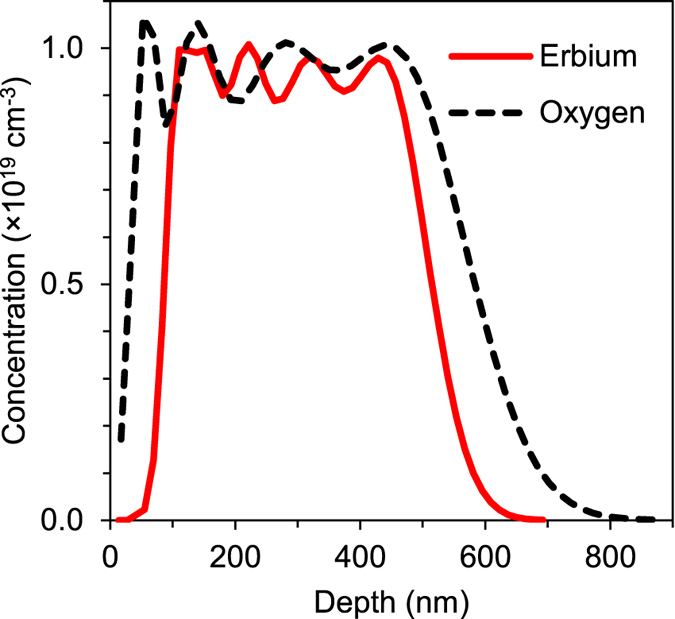
Erbium and oxygen carrier concentration profiles. Simulation results of Er and O carrier concentration profiles obtained by SUSPRE^60^. Different dopant doses and energies, listed in Table 1, were used to achieve equal ratio of erbium and oxygen.

A range of samples with different combinations of erbium and oxygen volume concentrations were used in this study (see Methods section). Figure 1 shows simulation results of implant concentration profiles for the sample implanted to 10^19^ Er cm^−3^ and O:Er ratio of 1. Both erbium and oxygen exhibit a flat profile in the range of 100 nm to 450 nm along the depth of the implants.

**Figure 2:**
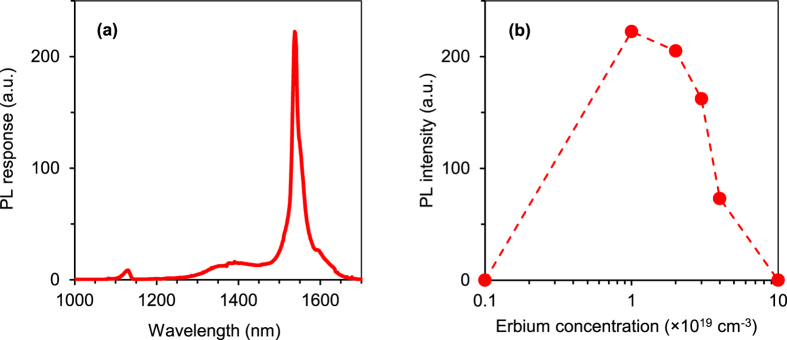
Photoluminescence × Er concentration. (**a**) PL spectrum, measured at 80 K, for an SOI sample implanted to 10^19^ Er cm^−3^ and O:Er ratio equal to 1; (**b**) PL intensity at 1.54 μm, measured at 80 K, for SOI samples of different Er volume concentrations and O:Er ratio equal to 1.

Figure 2(a) shows a typical PL spectrum measured at 80 K for an SOI sample implanted to 10^19^ Er cm^−3^ and O:Er ratio equal 1. The spectrum shows a strong Er peak at 1.54 μm due to the Er^3+^ internal transition from the ^4^I_13/2_ excited state to the ^4^I_15/2_ ground state and a weak emission around 1.1 μm due to the silicon band-edge. Figure 2(b) shows the PL intensity at 1.54 μm, measured at 80 K, as a function of the Er volume concentration, for SOI samples with O:Er ratio equal 1. The PL intensity at 1.54 μm shows a monotonic increase as more erbium is introduced, up to a maximum intensity at a concentration of 10^19^ cm^−3^. The PL intensity decreases as the Er and O doses are further increased due to a higher concentration of precipitates and defects that provide alternative non-radiative recombination routes that reduces the radiative recombination^35^.

**Figure 3:**
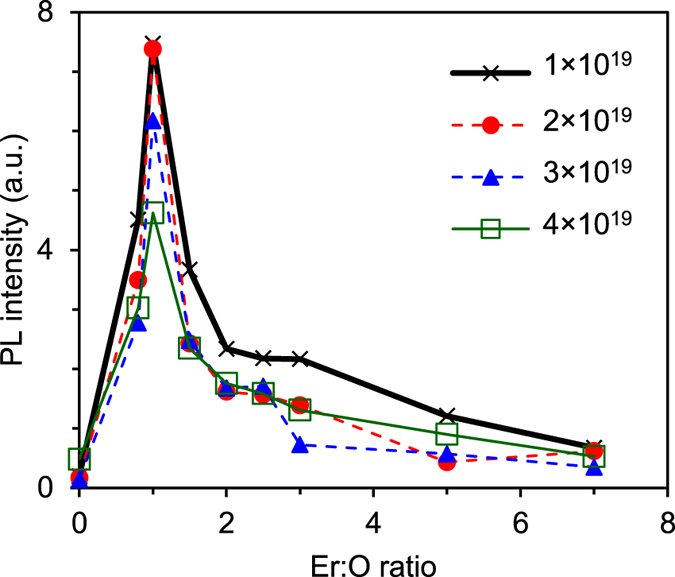
Photoluminescence × O:Er ratio. PL intensity at 1.54 μm, measured at 80 K, for SOI samples of different Er volume concentrations as a function of the O:Er ratio.

Figure 3 shows the 80 K PL intensity at 1.54 μm as a function of the O:Er ratio for SOI samples of different Er volume concentrations. The O:Er ratio equal to zero corresponds to samples implanted with Er only. Addition of oxygen clearly enhances the PL for all Er concentrations. The PL intensity increases dramatically with the addition of oxygen, reaches its maximum for an O:Er ratio of 1 and then drops sharply - a saturation regime is found when O:Er ratio is greater than 3. The 10^19^ Er cm^−3^ doped SOI sample exhibited a maximum increase in PL intensity overall which is 40 times higher than the PL intensity of the samples implanted with erbium only. For this sample we obtain a lower limit for the internal quantum efficiency of 0.22%. For O:Er ratios greater than 3 the PL enhancement drops considerably to ~5 times. The multimodal form of the PL intensity against O:Er ratio with a very sharply defined peak at an O:Er ratio of 1 clearly indicates that the singly oxygen coordinated Er^3+^ is in by far the most optically preferred configuration. The sharp reduction in the PL enhancement for higher O:Er ratios indicates that the different lattice configurations expected for multiply coordinated Er^3+^ lead to optically less favourable crystal field splittings.

The net enhancement of the maximum PL, as well as of the saturation regime, will depend on the residual oxygen concentration of the virgin, unprocessed substrates. The smaller enhancement previously reported in bulk silicon (less than 10 times)^30^,^35^,^36^,^46^,^48^ as compared to the SOI samples is therefore probably due to the high residual oxygen concentration found in bulk CZ silicon substrates, typically close to 10^18^ cm^−3^ [ref. 45], which would already lead to some PL enhancement without any additional oxygen implantation. The maximum SOI PL enhancement observed here occurs for equal oxygen and erbium volume concentrations, independent of the Er dose, suggesting that only a rather simple 1:1 Er:O complex is necessary for enhancement of the Er emission and that Er unpaired with O is only weakly emissive. Unpaired oxygen due to further O doping is found to be deleterious to the maximum emission enhancement. Oxygen codoping is predicted to increase the solubility of erbium in silicon and so increase the obtainable luminescence efficiency. The most stable configuration for the highest erbium solubility is, theoretically, the erbium atom in the tetrahedral interstitial site surrounded by six oxygen atoms^57^,^58^. This has led to a tendency to overdose with a significant excess of oxygen during implantation^36^ or growth^46^. However it is also predicted that alternative site configurations can alter the electronic states^57^ and so, in principle, the optical activity. More recent density functional computations^59^ have indicated two stable configurations, depending on the O:Er ratio (n): for n > 5 the tetrahedral interstitial site is again favoured but for n ≤ 2 the tetrahedral substitutional site is the most stable. Our results suggest that this site is also the most optically active of the two configurations and leads to the highest luminescence efficiency. This is attributed to a more favourable crystal field splitting in the case of the singly oxygen coordinated Er, which is in a tetrahedral substitutional site compared to that in the interstitial site favoured for higher oxygen coordination numbers.

**Figure 4:**
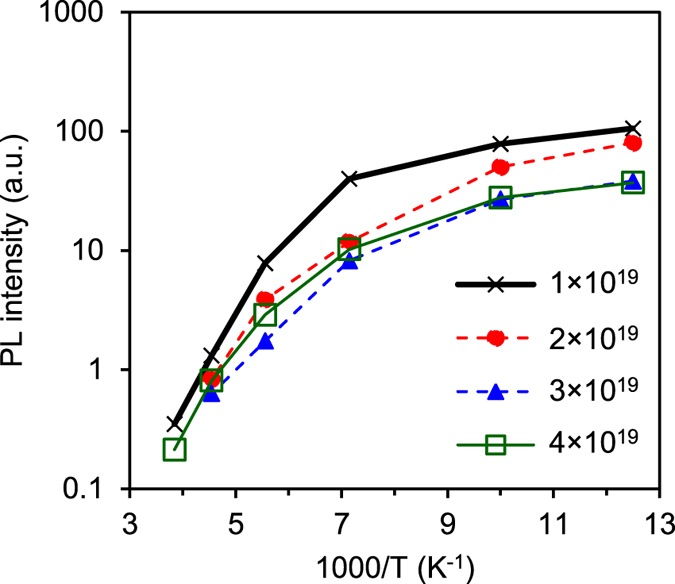
Photoluminescence × Temperature. Temperature dependence of the PL intensity at 1.54 μm for SOI samples of different Er concentrations and O:Er ratio of 1.

Figure 4 shows temperature dependence of the PL intensity at 1.54 μm for SOI samples of different Er volume concentrations and same O:Er ratio of 1. Photoluminescence quenching with temperature is observed and is largely independent of the Er dose. Previous PL results from Er:Si samples fabricated using the dislocation engineering approach^6^ have shown a reduction/elimination/reversal of the thermal quenching in both bulk silicon and SOI substrates^24^,^56^. The dislocation engineering approach uses the controlled introduction of dislocation loops, usually formed by boron implantation followed by thermal annealing, to provide carrier confinement and reduce or eliminate non-radiative recombination, thus enhancing the radiative recombination. This approach, combined with the optimized erbium and oxygen codoping, could offer a route to Er doped silicon emitters operating at higher temperatures.

In summary, we have designed, fabricated and characterized O:Er doped SOI samples at the wavelength of 1.54 μm. The results show that optical response is critically determined by the amount and ratio of erbium and oxygen present in the sample. The most intense photoluminescence peak is obtained in samples having equal ratio of erbium and oxygen. This indicates that the substitutional site for the singly coordinated case has the most favourable crystal field splitting. These data reveal very promising prospects for realization of light sources, optical amplifiers, silicon nanophotonics and other emerging applications in silicon.

## Methods

N-type SOI wafers (100) with resistivity of 10 Ωcm, top silicon layer of 2 μm and buried oxide layer of 2 μm were implanted to achieve Er volume concentrations in the 10^18^–10^20^ cm^−3^ range. Multiple implant erbium energies at 1.50, 1.00, 0.65, 0.40 and 0.25 MeV were used to provide an essentially uniform erbium profile to a depth of about 0.5 μm. The implanted Er doses and energies used to obtain a flat 10^19^ Er cm^−3^ volume concentration are listed in Table 1. Subsequently multiple energy implants of oxygen at 15, 45, 100 and 200 keV were made to match the erbium profile. The oxygen doses were chosen to correspond to O:Er ratios of 0.8, 1, 1.5, 2, 2.5, 3, 5 and 7 and are listed in Table 1 for the 10^19^ O cm^−3^ concentration. The other Er and O implanted doses can be obtained by direct multiplication of the values shown in Table 1 - the same implant energies were used for all samples. These implantation parameters, suggested by SUSPRE^60^, were used to suitably form a flat Er/O profile, thus ensuring good uniformity and matching of implant concentration along the depth of the implants. After the oxygen implantation, all samples were annealed in nitrogen ambient at 900 °C for 2 minutes to repair the lattice damage and to optically activate the Er/O.

Photoluminescence measurements were performed in the temperature range of 80–300 K across the 1.0–1.7 μm spectral region using a liquid-nitrogen-cooled Ge detector. Samples were mounted in a continuous flow liquid-nitrogen-cooled cryostat and excited by a 532 nm laser line at a power density of 100 mW/mm^2^. The quantum efficiency was obtained by direct comparison of the PL intensity with a calibrated standard sample measured under identical experimental conditions.

## Additional Information

**How to cite this article**: Lourenço, M. A. *et al*. Super-enhancement of 1.54 µm emission from erbium codoped with oxygen in silicon-on-insulator. *Sci. Rep.* **6**, 37501; doi: 10.1038/srep37501 (2016).

**Publisher's note:** Springer Nature remains neutral with regard to jurisdictional claims in published maps and institutional affiliations.

## Figures and Tables

**Table 1:** Implantation details.

Erbium		Oxygen	
Energy (MeV)	Dose (10^13^ cm^−2^)	Energy (keV)	Dose (10^13^ cm^−2^)
1.50	16.0	15	4.0
1.00	10.0	45	9.0
0.65	7.5	100	15.0
0.40	5.0	200	27.0
0.25	3.3		

Erbium and oxygen implanted doses and energies for the 1 × 10^19^ ion cm^−3^ volume concentration.
